# A Novel Hepatic Anti-Fibrotic Strategy Utilizing the Secretome Released from Etanercept-Synthesizing Adipose-Derived Stem Cells

**DOI:** 10.3390/ijms20246302

**Published:** 2019-12-13

**Authors:** Jae Hyun Han, Ok-Hee Kim, Sang Chul Lee, Kee-Hwan Kim, Jung Hyun Park, Jae Im Lee, Kyung Hee Lee, Ha-Eun Hong, Haeyeon Seo, Ho Joong Choi, Ji Hyeon Ju, Say-June Kim

**Affiliations:** 1 Department of Surgery, St. Vincent Hospital, College of Medicine, The Catholic University of Korea, Seoul 16247, Korea; gelasius@catholic.ac.kr; 2 Department of Surgery, Seoul St. Mary’s Hospital, College of Medicine, The Catholic University of Korea, Seoul 06591, Korea; ok6201@hanmail.net (O.-H.K.); kyunghee-03@nate.com (K.H.L.); hhe49@naver.com (H.-E.H.); searcx12@naver.com (H.S.); hopej0126@gmail.com (H.J.C.); 3 Catholic Central Laboratory of Surgery, College of Medicine, The Catholic University of Korea, Seoul 06591, Korea; keehwan@catholic.ac.kr; 4 Department of Surgery, Daejeon St. Mary’s Hospital, College of Medicine, The Catholic University of Korea, Seoul 34943, Korea; zambo9@catholic.ac.kr; 5 Department of Surgery, Uijeongbu St. Mary’s Hospital, College of Medicine, The Catholic University of Korea, Seoul 11765, Korea; lji96@catholic.ac.kr; 6 Department of Surgery, Eunpeong St. Mary’s Hospital, College of Medicine, The Catholic University of Korea, Seoul 03312, Korea; angle49@catholic.ac.kr; 7 Division of Rheumatology, Department of internal medicine, Seoul St. Mary’s Hospital, College of Medicine, The Catholic University of Korea, Seoul 06591, Korea; juji@catholic.ac.kr

**Keywords:** adipose-derived stem cells, etanercept, liver fibrosis, secretome, tumor necrosis factor-α (TNF-α)

## Abstract

Tumor necrosis factor-α (TNF-α)-driven inflammatory reaction plays a crucial role in the initiation of liver fibrosis. We herein attempted to design genetically engineered adipose-derived stem cells (ASCs) producing etanercept (a potent TNF-α inhibitor), and to determine the anti-fibrotic potential of the secretome released from the etanercept-synthesizing ASCs (etanercept-secretome). First, we generated the etanercept-synthesizing ASCs by transfecting the ASCs with mini-circle plasmids containing the gene insert encoding for etanercept. We subsequently collected the secretory material released from the etanercept-synthesizing ASCs and determined its anti-fibrotic effects both in vitro (in thioacetamide [TAA]-treated AML12 and LX2 cells) and in vivo (in TAA-treated mice) models of liver fibrosis. We observed that while etanercept-secretome increased the viability of the TAA-treated AML12 hepatocytes (*p* = 0.021), it significantly decreased the viability of the TAA-treated LX2 HSCs (*p* = 0.021). In the liver of mice with liver fibrosis, intravenous administration of the etanercept-secretome induced significant reduction in the expression of both fibrosis-related and inflammation-related markers compared to the control group (all *P*s < 0.05). The etanercept-secretome group also showed significantly lower serum levels of liver enzymes as well as pro-inflammatory cytokines, such as TNF-α (*p* = 0.020) and IL-6 (*p* = 0.021). Histological examination of the liver showed the highest reduction in the degree of fibrosis in the entanercept-secretome group (*p* = 0.006). Our results suggest that the administration of etanercept-secretome improves liver fibrosis by inhibiting TNF-α-driven inflammation in the mice with liver fibrosis. Thus, blocking TNF-α-driven inflammation at the appropriate stage of liver fibrosis could be an efficient strategy to prevent fibrosis.

## 1. Introduction

Liver fibrosis is characterized by cell death of hepatocytes, hepatic inflammation, and activation of hepatic stellate cells (HSCs). Tumor necrosis factor-α (TNF-α) is one of the predominant cytokines involved in all these steps leading to liver fibrosis. It is produced by the activated macrophages and other liver cells in response to liver injuries. TNF-α was found to be significantly increased in the damaged livers following bile duct ligation (BDL) [1]. BDL did not induce significant liver injury or liver fibrosis in TNF-α^−/−^ mice [2] and TNF receptor 1 (TNFR1)^−/−^ mice, suggesting that TNF-α is significantly involved in the pathophysiology following BDL [1,2,3]. Consistently, it was reported that the mice with non-alcoholic steatohepatitis (NASH) lacking the TNF receptor showed reduced steatosis, inflammation, and fibrosis [4]. Also, the serum level of TNF-α was found to be increased in NAFLD patients [5]. On the contrary, there are also reports that TNF-α protects the liver from damage [6], promotes liver regeneration [7], and ameliorates liver fibrosis by inhibiting the expression of collagen type 1 alpha 1 chain (COL1A1) in HSCs [8,9]. Therefore, the role of TNF-α in the progression of liver fibrosis should be determined more carefully.

**Figure 1:**
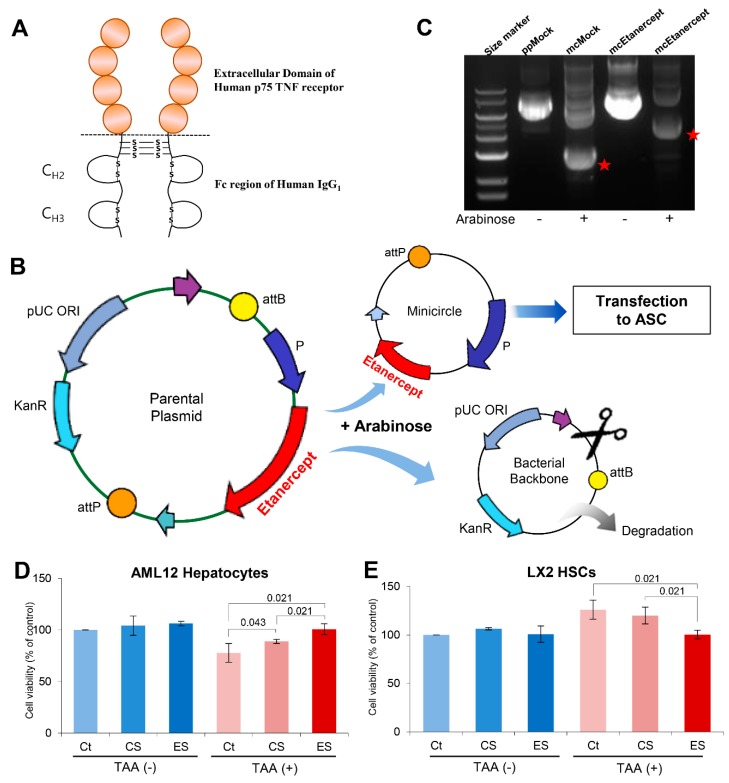
Generation of etanercept-synthesizing ASCs and in vitro validation. (**A**) Structure of etanercept. Etanercept is a fusion protein composed of the binding between the p75 region of TNF receptor 2 and the Fc portion of immunoglobulin. (**B**) Agarose gel image showing the successful generation of the mini-circle plasmids encoding for etanercept. Red stars represent control minicircle DNA and etanercept-encoding minicircle DNA, respectively. (**C**) Generation of the mini-circle plasmid encoding for etanercept. Arabinose treatment to the parental plasmid results in the release of mini-circle plasmids encoding for etanercept. (Source from https://www.biocat.com/genomics/cloning-kits/minicircle-dna-vector-technology-non-integrative-sustained-expression). (**D**) Effects of the etanercept-secretome on the cell viability of AML12 hepatocytes. The individual secretome treatments, especially etanercept-secretome, induced significantly higher cell viability of AML12 hepatocytes 24 h after treatment. (**E**) Effects of etanercept-secretome on the cell viability of LX2 HSCs. Etanercept-secretome significantly decreased the cell viability of TAA-treated LX2 cells, especially 48 h after treatment. Values are presented as mean ± standard deviation of three independent experiments. Abbreviations: Ct, control; CS, control secretome. ES, etanercept-secretome; TAA, thioacetamide; HSC, hepatic stellate cell; mcEtanercept, etanercept-encoding minicircle DNA; mcMock, minicircle DNA without encoding etanercept; ppMock, parenteral plasmid without encoding etanercept; TNF-α, tumor necrosis factor-α.

Etanercept is a biopharmaceutical that is used to treat autoimmune diseases. A variety of autoimmune diseases are caused by an excessive immune response that is predominantly mediated by TNF-α. Etanercept can ameliorate autoimmune diseases by acting as a TNF-α inhibitor. Etanercept has been approved by the US FDA for the treatment of a number of autoimmune diseases, including rheumatoid arthritis, juvenile idiopathic arthritis and psoriatic arthritis, plaque psoriasis, and ankylosing spondylitis [10]. Etanercept is a fusion protein that is formed due to the binding between the p75 region of TNF receptor 2 and the Fc portion of immunoglobulin (Figure 1A) [10,11,12]. Although having higher therapeutic potential, etanercept can be categorized as an expensive medicine because it is produced through a series of recombination and purification processes.

TNF-α-driven inflammatory reaction plays a crucial role in the initiation of liver fibrosis [5,13]. It was reported that the antifibrogenic effect of etanercept attenuated the progression of liver cirrhosis induced by thioacetamide in rats [14]. Thus, we attempted to design genetically engineered adipose-derived stem cells (ASCs) to produce etanercept in order to ameliorate liver fibrosis using the secretome obtained from these etanercept-synthesizing ASCs. ASCs exhibit immunomodulatory, anti-inflammatory, and tissue repairing functions through secretomes. Moreover, if ASCs were equipped with the ability to synthesize etanercept, it is expected that they could not only strongly suppress the acute phase of liver fibrosis but also reduce the burden of high cost of etanercept. Secretome refers to the total set of molecules secreted or surface-shed by stem cells [15,16]. The reason why we decided to use the secretome instead of etanercept-synthesizing ASCs is to overcome certain limitations associated with stem cell therapy. Despite numerous potential benefits, stem cells have several limitations in their clinical application. One of the limitations is the short-term retention of stem cells after transplantation as most of stem cells are lost in just a few days [17,18,19,20]. Furthermore, stem cells can potentially transform into malignant tumors [21,22]. Meanwhile, numerous studies have shown that the stem-cell secretome has similar therapeutic potential as stem cells because the main working mechanism of stem cells is secretome-mediated [23,24,25,26,27,28,29,30]. In this experiment, we expected that etanercept-synthesizing ASCs would release a variety of anti-inflammatory and immunomodulatory materials along with etanercept, the sum of which could exert the strengthened anti-fibrotic potential during the process of liver fibrosis.

## 2. Results

### 2.1. Attainment of Etanercept-Secretome from Etanercept-Synthesizing ASCs

The gene encoding etanercept was inserted into the parental plasmid (mcTNFR2). Subsequently, we treated the parental plasmid with arabinose for the release of mini-circle plasmids encoding for etanercept (Figure 1B). The successful generation of the mini-circle plasmids encoding etanercept was demonstrated using agarose gel electrophoresis (Figure 1B). These mini-circles were then transfected into ASCs to produce etanercept-synthesizing ASCs (etanercept-ASCs) (Figure 1C). We finally obtained the secretome (etanercept-secretome) from these etanercept-ASCs after a series of processes, including centrifugation and filtering, all of which are mentioned in the methods section. Herein, control secretome refers to the secretome obtained from empty vector-transfected ASCs, and etanercept-secretome refers to the secretome obtained from etanercept-synthesizing ASCs.

We examined the effect of each secretome sample (control secretome and etanercept-secretome) on the viability of AML12 hepatocytes and LX2 HSCs, respectively. TAA is a hepatoxin that can simulate the condition of liver damage or fibrosis when it is used in liver cells. We thus divided each kind of cells (AML12 and LX2 cells) into two groups, and treated with and without TAA. In AML12 hepatocytes treated with TAA, the cell viability was improved in the control secretome (*p* = 0.43) and etanercept-secretome groups (*p* = 0.021) compared to the control group (Figure 1D). When comparing between control secretome and etanercept-secretome groups, etanercept-secretome group exhibited significantly higher viability than the control secretome group (*p* = 0.021). In LX2 cells treated with TAA, the cell viability was significantly reduced in the etanercept-secretome group compared to the control group (*p* = 0.021) (Figure 1E). When comparing between control secretome and etanercept-secretome groups, etanercept-secretome group exhibited significantly lower viability than control secretome group (*p* = 0.021). Taken together, it appeared that while etanercept-secretome increased the cell viability of AML12 hepatocytes, it significantly decreased the cell viability of TAA-treated LX2 cells. These results suggest that whereas etanercept-secretome could promote cell viability of normal hepatocytes, it could significantly lower the viability of HSCs during the process of liver fibrosis.

### 2.2. Effects of Etanercept-Secretome on the Protein Expression in HSCs in Vitro

**Figure 2:**
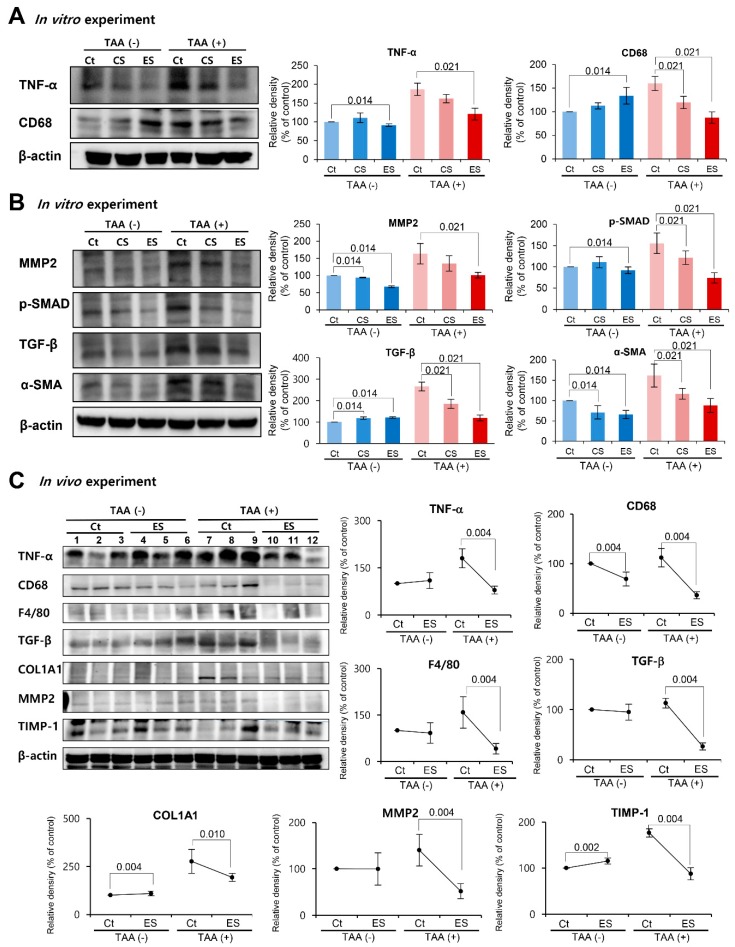
Effects of etanercept-secretome on the expression of proteins in the livers of mice with liver fibrosis. (**A**) Effects of etanercept-secretome on the expression of inflammation-related proteins (TNF-α and CD68) in LX2 HSCs. In the LX2 HSCs with TAA treatment, the expression levels of TNF-α and CD68 were significantly lower in etanercept-secretome group than in control group. (**B**) Effects of etanercept-secretome on the expression of fibrosis-related (MMP2, p-SMAD, TGF-β, and α-SMA) in LX2 HSCs. In the LX2 HSCs with TAA treatment, the expression levels of these fibrosis-related proteins were significantly lower in etanercept-secretome group than in control group. (**C**) Western blot analysis of the livers obtained from the mice with liver fibrosis following individual treatments (control secretome and etanercept-secretome). Expression levels of inflammation- (TNF-α, CD68, and F4/80) and fibrosis-related proteins (TGF-β, COL1A1, MMP2, and TIMP-1) in the liver specimens were investigated following individual treatments. Relative densities of individual markers had been quantified using Image J software and then were normalized to that of β-actin in each group. The values are presented as mean ± standard deviation of three independent experiments. Abbreviations: α-SMA, alpha smooth muscle actin; COL1A1, collagen type 1 alpha1; CS, secretome obtained from empty vector-transfected ASCs; Ct, control; ES, etanercept-secretome (the secretome obtained from etanercept-synthesizing ASCs); HSC, hepatic stellate cell; MMP2, metalloproteinases-2; Sal, saline injection group; TNF-α, tumor necrosis factor-α; TIMP-1, tissue inhibitor of metalloproteinases-1; TGF-β, transforming growth factor-β; TNF-α, tumor necrosis factor-α.

Next, we examined the effects of each of the etanercept-secretome on the expression of inflammation-related proteins (TNF-α and CD68) in LX2 HSCs with or without treatment with TAA. In the HSCs without TAA treatment, the two groups (control and etanercept-secretome groups) showed variable alternations in the expression of these inflammation-related proteins. However, in the LX2 HSCs with TAA treatment, the expression levels of these inflammation-related proteins were significantly lower in etanercept-secretome group than in control group (all *P*s < 0.05) (Figure 2A).

Subsequently, we examined the effects of each of the etanercept-secretome on the expression of fibrosis-related proteins (MMP2, p-SMAD, TGF-β, and α-SMA) in LX2 HSCs with or without treatment with TAA. In the HSCs without TAA treatment, the two groups showed variable alternations in the expression of the fibrosis -related proteins. However, in the LX2 HSCs with TAA treatment, the expression levels of these fibrosis-related proteins were significantly lower in etanercept-secretome group than in control group (all *P*s < 0.05) (Figure 2B).

### 2.3. Effects of the Etanercept-Secretome in an In Vivo Liver Fibrosis Model

To validate the in vivo effects of etanercept-secretome, we generated in vivo model of liver fibrosis by subcutaneous injection of TAA (200 mg/kg, three times a week for 8 weeks) into experimental mice [31,32,33,34,35,36]. Subsequently, control mice (*n* = 30) and TAA-treated mice (*n* = 30) received four injections (two times a week during 7th and 8th week of TAA treatment) of 0.1 mL normal saline (*n* = 10), 0.1mL control secretome (*n* = 10), and 0.1mL etanercept-secretome (*n* = 10), respectively. We collected the serum samples and the liver specimens of euthanized mice on the 7th day after initial injection.

We first performed western blot analysis for the determination of the expression of inflammation- and fibrosis-related markers in the liver specimens (Figure 2C). In the control mice without TAA treatment, the two groups (control and etanercept-secretome groups) showed no significant difference in the expression of the inflammation-related proteins (TNF-α, CD68, and F4/80). However, in the mice with TAA-induced liver fibrosis, the expression levels of these inflammation-related proteins were significantly lower in etanercept-secretome group than in saline group (all *P*s < 0.05). Subsequently, we examined the effects of the etanercept-secretome on the expression of fibrosis-related proteins (TGF-β, COL1A1, MMP2, and TIMP-1) in the liver specimens. In the control mice, the two groups showed no significant difference in the expression of the fibrosis-related proteins. However, in the mice with liver fibrosis, the expression levels of these fibrosis-related proteins were significantly lower in etanercept-secretome group than in saline group (all *P* < 0.05). The control secretome group showed the values between the saline and etanercept-secretome groups in the expression of these markers.

**Figure 3:**
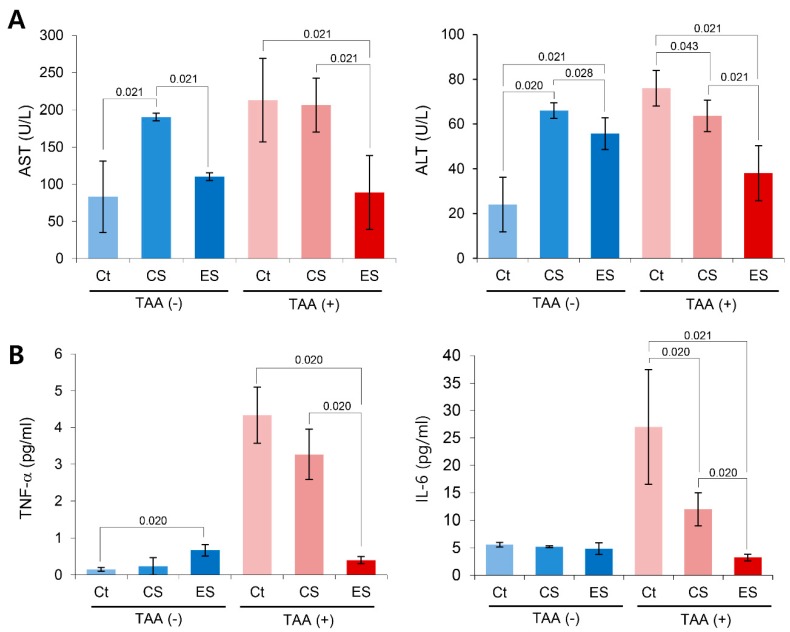
Anti-fibrotic effects of the etanercept-secretome in an in vivo model of liver fibrosis. (**A**) Effects of etanercept-secretome on the serum levels of liver enzymes. Of mice with two kinds of secretome treatment (control secretome and etanercept-secretome), etanercept-secretome group showed the significantly lower AST and ALT than did control secretome group. (**B**) Effects of etanercept-secretome on the serum levels of pro-inflammatory cytokines (TNF-α and IL-6). Etanercept-secretome group showed the lowest serum levels of TNF-α and IL-6 of all. The values are presented as mean ± standard deviation of three independent experiments. Abbreviations: ALT, alanine transaminase; AST, aspartate transaminase; COL1A1, collagen type 1 alpha1; CS, secretome obtained from empty vector-transfected ASCs; Ct, control; ES, etanercept-secretome (secretome obtained from etanercept-synthesizing ASCs); Sal, saline injection group; TNF-α, tumor necrosis factor-α.

Next, we compared the serum levels of the liver enzymes in each group 7 days post-injection (Figure 3A). Mice with etanercept-secretome treatment showed the significantly lower serum levels of AST (*p* = 0.021) and ALT (*p* = 0.021) than the control mice (saline group). For determining the effects of each secretome on the systemic inflammation, we compared the serum levels of pro-inflammatory cytokines, such as TNF-α and IL-6, in each group. It was found that etanercept-secretome group showed the significantly lower levels of TNF-α (*p* = 0.020) and IL-6 (*p* = 0.021) than those of TAA-treated mice (Figure 3B).

### 2.4. Histological Alternation of Liver Specimens after Injection of Etanercept-Secretome

**Figure 4:**
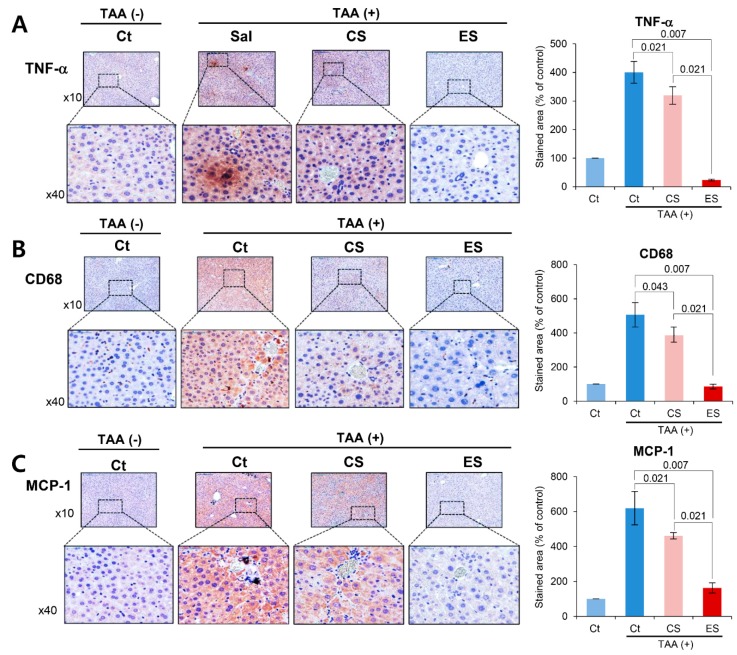
Immunohistochemistry of inflammatory markers in the liver specimens. (**A**) CD68 immunohistochemistry of the livers in each group. The etanercept-secretome group showed the significant lower expression of CD68. (**B**) TNF-α immunohistochemistry of the livers in each group. The etanercept-secretome group showed the significant lower expression of TNF-α. (**C**) MCP-1 immunohistochemistry of the livers in each group. The etanercept-secretome group showed the significant lower expression of MCP-1. The percentages of immunoreactive areas were measured using NIH image J and expressed as values relative to those from normal livers. Values are presented as mean ± standard deviation of three independent experiments. Abbreviations: CS, secretome obtained from empty vector-transfected ASCs; Ct, control; ES, etanercept-secretome (secretome obtained from etanercept-synthesizing ASCs); MCP-1, monocyte chemoattract protein-1; Sal, saline injection group; TNF-α, tumor necrosis factor-α.

We compared the histological changes of the livers in each group on post-injection day 7. We first performed the immunohistochemistry of inflammation-related markers, such as TNF-α, CD68, and MCP-1 (Figure 4A–C). Percentages of immunoreactive areas for TNF-α, CD68, and MCP-1 were quantified using NIH image J and expressed as relative values to those in normal livers. Although the immunoreactive areas were increased after TAA treatment, secretome treatments significantly reduced them (all *P*s < 0.05), and of the two secretome groups, the etanercept-secretome group showed the smallest immunoreactive areas (*p* = 0.020).

**Figure 5:**
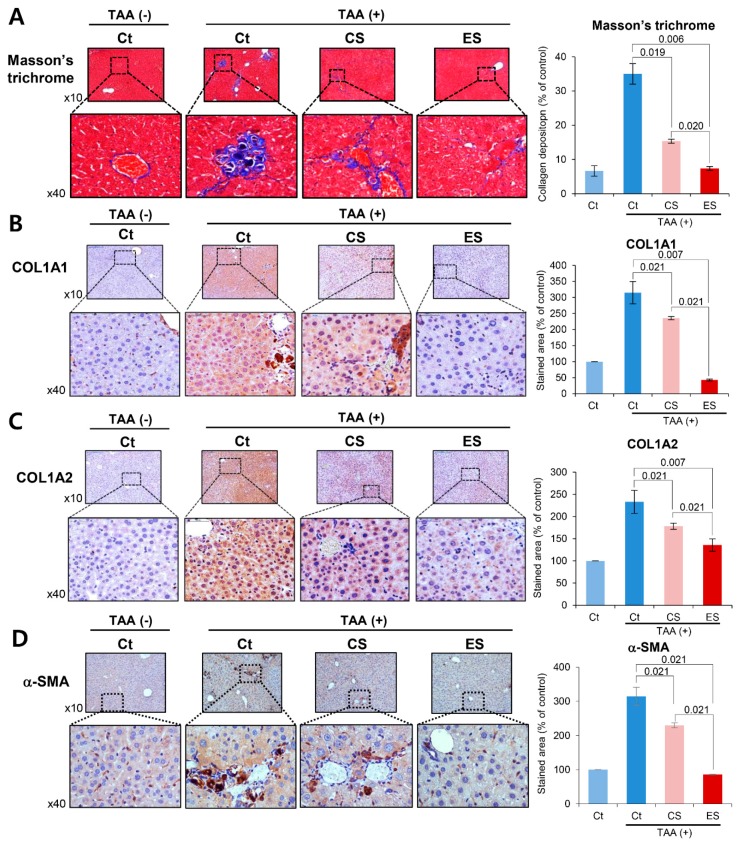
Special histological stains reflecting the degree of fibrosis in the livers in each group. (**A**) Masson’s trichrome staining of each group on the seventh day post-injection. Percentages of fibrotic areas were estimated using NIH image J. (**B**,**C**) COL1A1 and COL1A2 immunohistochemistry of the livers on the seventh day post-injection for the determination of the collagen content in each group. (**D**) α-SMA immunohistochemistry of the livers on the seventh day post-injection for the indirect localization of activated HSCs in each group. Percentages of immunoreactive areas were estimated using NIH image J. The values are presented as mean ± standard deviation of three independent experiments. Abbreviations: α-SMA, alpha smooth muscle actin; COL1A1, collagen type 1 alpha1; COL1A2, collagen type 1 alpha2; CS, secretome obtained from empty vector-transfected ASCs; Ct, control; ES, etanercept-secretome (secretome obtained from etanercept-synthesizing ASCs); Sal, saline injection group.

**Figure 6:**
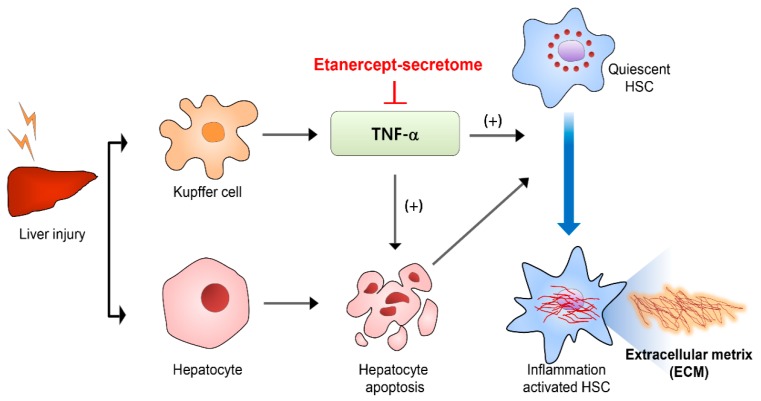
Suggested mechanism of action of etanercept-secretome. Activated HSCs cause liver fibrosis by accumulating collagen and other extracellular matrix components. Hepatocyte apoptosis is one of the representative events leading to the activation of HSCs. Pro-fibrogenic response occurs when the HSCs engulf the apoptotic bodies that are ejected from the hepatocytes as a result of hepatocyte apoptosis. TNF-α is predominantly released from the Kupffer cells in the liver, and plays a crucial role in inducing apoptosis of hepatocytes. Etanercept-secretome appears to ameliorate liver fibrosis, principally by blocking the action of TNF-α. Abbreivations: HSC, hepatic stellate cell: TNF-α, tumor necrosis factor-α.

In Masson’s trichrome stains, percentages of fibrotic areas were estimated using NIH image J. Although the fibrotic areas were increased after TAA treatment, secretome treatments significantly reduced them (all *P*s < 0.05), and of the two secretome groups, the etanercept-secretome group showed the smallest fibrotic area (*p* = 0.020) (Figure 5A). Subsequently, we performed COL1A1 and COL1A2 immunohistochemistry of the livers for the determination of the collagen content in each group (Figure 5B,C). It was found that secretome treatments significantly reduced the collagen content (all *P*s < 0.05), and of the two secretome groups, the etanercept-secretome group showed the lowest amount of collagen in the liver (*p* = 0.021). Finally, we performed α-SMA immunohistochemistry of the livers for the indirect localization of activated HSCs in each group (Figure 5D). It was found that secretome treatments significantly reduced the expression of α-SMA (all *P*s < 0.05), and of the two secretome groups, the etanercept-secretome group showed the lowest expression of α-SMA (*p* = 0.021). Taken together, our results showed that etanercept-secretome can potentially ameliorate inflammation as well as fibrosis in the mice with liver fibrosis. Based on the results of our experiment, we provided the possible mechanism of action of etanercept-secretome in the livers of mice with liver fibrosis (Figure 6).

## 3. Discussion

TNF-α is released during the inflammatory phase of liver fibrosis, and plays a decisive role in the progression of liver fibrosis by activating HSCs to secrete fibrogenic materials. In this study, we constructed engineered ASCs that can synthesize etanercept (a potent TNF-α inhibitor), and determined the anti-inflammatory and anti-fibrotic potential of the secretome released from etanercept-synthesizing ASCs. For the in vivo validation of the effects of etanercept-secretome, we intravenously administrated control- and etanercept-secretome, respectively, to the mice with liver fibrosis. Injection of etanercept-secretome led to the significant reduction in the expression levels of both the inflammation- and fibrosis-related proteins in the livers compared to the injection of control secretome. Injection of etanercept-secretome also resulted in the significantly lower serum levels of liver enzymes as well as pro-inflammatory cytokines, such as TNF-α and IL-6, in the mice with liver fibrosis. Histological examination of the liver specimens validated that the etanercept-secretome group also exhibited the lowest degree of fibrosis in the liver. Taken together, our results suggest that the injection of etanercept-secretome protects the liver against inflammatory and fibrotic damages by inhibiting TNF-α-driven inflammation during liver fibrosis.

Interestingly, etanercept-secretome increased the viability of hepatocytes but decreased the viability of HSCs. ASCs basically have anti-inflammatory, immunomodulatory, and tissue-repairing functions, all of which are principally mediated by secretome. Because of this mechanism, secretome administration to the TAA-treated hepatocytes appears to increased cell viability. However, administration of etanercept-secretome to the TAA-treated LX2 HSCs reduced cell viability. This could be attributed to the effect of the etanercept contained in the etanercept-secretome. TAA-treated HSCs are supposed to be strongly activated by TNF-α and thereby secrete various fibrogenic materials. Etanercept-secretome could reduce the activity of HSCs due to the potent anti-inflammatory effects of etanercept, resulting in decreased cell viability. Although statistically insignificant, secretome treatment also reduced the cell viability of HSCs, suggestive of anti-fibrogenic effects of secretome itself. Indeed, several studies have reported the antifibrogenic effects of mesenchymal stem cells or their secretome in the model of liver fibrosis [37,38,39,40,41,42]. Since liver fibrosis process is initiated by inflammation, it appears that secretome reduces the viability of HSCs through anti-inflammatory activities.

Our study shows that etanercept-secretome has potent anti-inflammatory activity. In the in vivo model, the administration of etanercept-secretome not only suppressed TNF-α expression but also significantly reduced the expression of pro-inflammatory mediators CD68 and MCP-1 in the liver specimens. Moreover, the administration of etanercept-secretome resulted in the significant reduction of serum concentration of TNF-α and IL-6. It appears that such strong anti-inflammatory effect could have led to the subsequent anti-fibrogenic consequences of liver specimens. This suggests that, since inflammation accounts for a considerable proportion in the process of liver fibrosis, suppressing the inflammation at the appropriate timing could lead to the improvement of liver fibrosis.

Quiescent HSCs are activated by several substances released during liver parenchymal injury. Activated HSCs cause liver fibrosis by accumulating collagen and other extracellular matrix components. Hepatocyte apoptosis is one of the representative events leading to the activation of HSCs. Several studies have validated that a pro-fibrogenic response occurs when the HSCs engulf the apoptotic bodies that are ejected from the hepatocytes [43,44]. Out of a variety of cytokines and growth factors involved in this process, TNF-α is predominantly released from the activated macrophages in the liver, and binds to TNF-receptor-1 in the neighboring cells [5,13,45,46]. As a result, hepatocyte apoptosis occurs, and the HSCs initiate a pro-fibrogenic response immediately after recognizing hepatocyte apoptosis by engulfing the materials from apoptotic hepatocytes. In this study, we successfully ameliorated liver fibrosis by blocking the function of TNF-α by etanercept-secretome, and hence, demonstrated the significant role of TNF-α in the process of liver fibrosis.

There are several limitations of this study. This study did not provide a direct comparison between the etanercept and etanercept-secretome groups. We anticipate that the etanercept-secretome group would have stronger anti-fibrogenic effects than etanercept alone due to not only the expression of etanercept but also the release of various tissue-repairing factors derived from ASCs. In order to clearly demonstrate that this novel strategy may be more effective than etanercept, additional experiments are required to analyze the differences between etanercept-secretome and etanercept or another TNF-α blocker (such as a neutralizing antibody), which we planned to perform in the further study. In addition, this study did not provide accurate information on the cause of the superior anti-fibrogenic effects of the etanercept-secretome group. The accurate answer for this would be provided by component analysis between control secretome and etanercept-secretome using liquid chromatography–mass spectrometry.

In conclusion, we showed that the use of etanercept-secretome is capable of considerably ameliorating liver fibrosis by blocking TNF-α because TNF-α plays a crucial role in the inflammatory response in the liver fibrosis process. Etanercept-secretome refers to the total collection of secretory materials released from etanercept-synthesizing ASCs that are supposed to contain etanercept as well as the other components of materials shed from the ASCs. Our in vitro and in vivo experiments showed that etanercept-secretome exhibits stronger anti-inflammatory and anti-fibrotic effects than control secretome, which ultimately led to the higher amelioration of liver fibrosis. We thus believe that blocking TNF-α-driven inflammation at the appropriate stage of liver fibrosis could be an efficient strategy to prevent fibrosis. In addition, as the etanercept-secretome encompassed the effects of etanercept along with the other components of the secretome produced by etanercept-synthesizing ASCs, it could be more suitable for clinical application than etanercept alone considering effectiveness and economic aspects.

## 4. Materials and Methods

### 4.1. Cell Culture

Human ASCs were kindly donated by Hurim BioCell Co. Ltd. (Seoul, Republic of Korea) (IRB number 700069-201407-BR-002-01). ASCs were plated into culture flask in low-glucose Dulbecco’s modified Eagle’s medium (DMEM; Thermo Fisher Scientific, Carlsbad, CA, USA) supplemented with 10% FBS (Thermo Fisher Scientic), 100 U/mL of penicillin (Thermo Fisher Scientic), and 0.1 mg/mL of streptomycin (Thermo Fisher Scientic). The AML12 mouse hepatocyte cell line was obtained from American Type Culture Collection (ATCC; Manassas, VA, USA). AML12 cells were maintained in DMEM/F12 (Dulbecco’s modified Eagle medium/Ham’s F-12; Thermo Fisher Scientic). The medium was supplemented with 10% FBS (fetal bovine serum; GibcoBRL, Calsbad, CA), 1% antibiotics (Thermo Fisher Scientic), 1×ITS supplement (Insulin-Transferrin-Selenium-G supplement; Invitrogen, Calsbad, CA), and 40 ng/mL dexamethasone (Sigma-Aldrich, St. Louis, MO, USA). The LX-2 human stellate cells were kindly donated by Dr. Won-il Jeong in KAIST Biomedical research of Korea. LX-2 cells were maintained in DMEM (Dulbecco’s modified Eagle medium; Thermo Fisher Scientic, Carlsbad, CA). The medium was supplemented with 10% FBS (fetal bovine serum; GibcoBRL, Calsbad, CA), 1% antibiotics (Thermo Fisher Scientic). Cells were incubated at 37 °C in humidified chamber containing 5% carbon dioxide.

### 4.2. Generation of Etanercept-Synthesizing ASCs

The minicircles (mcMock and mcTNFR2) were kindly donated by Dr. Ji Hyeon J in the Catholic University of Korea [11]. Briefly, ZYCY10P3S2T competent cells transformed with the parental plasmids were grown overnight at 37 °C in Terrific Broth containing 50 μg/mL kanamycin. A single colony was grown for 8 h in Luria broth containing kanamycin. The cultures were combined with Luria broth containing 0.02% arabinose and incubated at 30 °C for 5 h. Minicircle DNA was isolated using the DNA-midi ^TM^ GT plasmid DNA purification kit (Intron biotechnology, Seongnam, Republic of Korea). 5.0 × 10^5^ ASCs were transfected with the minicircle vectors using Lipofectamine 2000 reagent (Invitrogen, Carlsbad, CA, USA) following the manufacturer’s instructions. ASCs (5 × 10^5^ cells) were transfected with mcTNFR2 to generate etanercept-secretome.

### 4.3. Attainment of Secretome

ASCs were grown in a 100 mm cell dishes (Corning Glass Works, Corning, NY, USA). After reaching 70–80% confluence, 1.0 × 10^6^ ASCs were cultured in 5 mL serum-free low-glucose DMEM for 48 h. Therefore, to obtain 0.2mL amount of secretome from 1.0 × 10^6^ ASCs, the conditioned media were concentrated 25-fold using ultra filtration units with a 3-kDa molecular weight cutoff (Amicon Ultra-PL 3; Millipore, Bedford, MA, USA). We then injected 0.1 mL amount of secretome per mouse. This means that one mouse is injected with the secretome obtained from 5 × 10^5^ ASCs. In this study, control secretome refers to the secretome obtained from empty vector-transfected ASCs, and etanercept-secretome refers to the secretome obtained from etanercept-synthesizing ASCs.

### 4.4. Cell Proliferation Assay

Cell viability of LX-2 HSCs and AML12 mouse hepatocytes were evaluated using EZ-Cytox Cell Proliferation Assay kit (Itsbio, Seoul, Republic of Korea) according to the manufacturer’s instructions.

### 4.5. Design of Animal Study

We used five-week male BALB/c mice (Orient Bio, Seongnam, Korea) in this study. Animal studies were carried out in compliance with the guidelines of the Institute for Laboratory Animal Research, Korea (IRB no. CUMC-2018-0331-01,4 Deceember 2018). We then compared the effects of the etanercept-secretome in an in vivo model of TAA-induced hepatic fibrosis model. The in vivo model of liver fibrosis was generated by subcutaneous injection of TAA (200 mg/kg, three times a week for 8 weeks) into experimental mice. Subsequently, control mice (*n* = 30) and TAA-treated mice (*n* = 30) received four injections (two times a week during seventh and eighth week of TAA treatment) of 0.1 mL normal saline (*n* = 10), 0.1mL control secretome (*n* = 10), and 0.1mL etanercept-secretome (*n* = 10), respectively. We collected the serum samples and the liver specimens on the seventh day after euthanizing mice.

### 4.6. Western Blot Analysis

LX2 cells and liver specimens obtained from mice were lysed using the EzRIPA Lysis kit (ATTO Corporation; Tokyo, Japan), and quantified by Bradford reagent (Bio-Rad, Hercules, CA, USA). Proteins were visualized by western blot analysis using the primary antibodies (1:1000 dilution) from Cell Signaling Technology (Beverly, MA, USA) and then with HRP-conjugated secondary antibodies (1:2000 dilution) from Vector laboratories (Burlingame, CA, USA). Primary antibodies included the antibodies against TNF-α (Tumor necrosis factor alpha), CD68 (Cluster of Differentiation 68), p-SMAD, F4/80, COL1A1 (Collagen type 1 alpha 1 chain), TGF-β (transforming growth factor-β), α-SMA (alpha-smooth muscle actin), TIMP-1 (Metallopeptidase inhibitor 1), MMP2 (Matrix Metallopeptidase-2), and β-actin. Specific immune complexes were detected using the Western Blotting Plus Chemiluminescence Reagent (Millipore, Bedford, MA, USA).

### 4.7. Serology Test and ELISA

Blood samples that had been collected from each mouse underwent centrifugation for 10 min at 9500 g for the attainment of serum. We measured the concentrations of markers for liver injury, such as aspartate transaminase (AST) and alanine transaminase (ALT), using an IDEXX VetTest Chemistry Analyzer (IDEXX Laboratories, Inc., Westbrook, ME, USA). The concentrations of mouse IL-6 and tumor necrosis factor-α (TNF-α) were measured by sandwich enzyme-linked immunosorbent (ELISA) assay (Biolegend, San Diego, CA, USA) according to the manufacturer’s instructions.

### 4.8. Immunohistochemistry and Masson’s Trichrome Staining

For immunohistochemical analysis, formalin-fixed, paraffin-embedded tissue sections were deparaffinized, rehydrated in an ethanol series, and subjected to epitope retrieval using standard procedures. Antibodies against of COL1A1 (working dilution 1:100), COL1A2 (1:100), CD68 (1:500), TNF-α (1:150), and monocyte chemoattract protein-1(MCP-1, 1:200) (all from Cell Signaling Technology, MA) were used for immunochemical staining. The samples were then examined under a laser-scanning microscope (Eclipse TE300; Nikon, Tokyo, Japan). Trichrome staining was performed using the Masson’s trichrome staining kit according to the manufacturer’s protocol (Polysciences, Warrington, PA, USA).

### 4.9. Statistical Analysis

All data were analyzed with SPSS 11.0 software (SPSS Inc., Chicago, IL, USA), and are presented as mean ± standard deviation (SD). Statistical comparison among groups was determined using Kruskal–Wallis test followed by Dunnett’s test as the post hoc analysis. Probability values of *p* < 0.05 were regarded as statistically significant.

## Abbreviations

TNF-α	Tumor necrosis factor-α
ASCs	ASCs adipose-derived stem cells
HSCs	Hepatic stellate cells
BDL	Bile duct ligation
